# Supplementary material for: Comparison of different Lunit INSIGHT CXR software versions when reading chest radiographs for tuberculosis
Source: PLOS Digit Health. 2025 Apr 15;4(4):e0000813. doi: 10.1371/journal.pdig.0000813 (PMC11999130; doi:10.1371/journal.pdig.0000813)
Supplement: Attachment — Submitted filename: PDIG-D-24-00271.pdf [file pdig.0000813.s001.pdf]

This manuscript addresses an important aspect of TB diagnostics in the context of evolving AI technologies. The study design is compact, and the findings are relevant to both research and clinical practice. However, several improvements in the clarity of the abstract, deeper methodological discussions, and expanded clinical implications would enhance the manuscript's overall impact.

1. The abstract needs to be rewritten. The objective of the study is not clearly stated and jumps directly into the contributions and results.
2. The phrase "performance characteristics were compared at selected cut-off thresholds" is unclear—briefly explain why these thresholds were chosen.
3. The introduction needs more details on why comparing CAD versions is important for clinical practice, especially in resource-constrained settings. While the shortage of radiologists is mentioned, explain how the findings could directly impact health outcomes.
4. Add more recent studies to highlight developments in CAD systems beyond the three mentioned systems (CAD4TB, INSIGHT CXR, qXR).
5. Add more recent studies on the clinical impact of CAD software in TB diagnostics within the field.
6. The limitations section should explain in more detail how using the Ultra test as a reference could bias the results. Although the issue of false positives in individuals with a past TB history is mentioned, expanding on the potential for this bias and its effect on the study's conclusions is necessary.
7. Highlight the need for prospective validation more clearly. The differences between study conditions and real-world settings should be expanded.
8. In the methods section, how does the current threshold calibration method (TDR or iterative score calibration) compare in terms of ease of implementation, costs, and time?
9. Provide further details on the anonymization of DICOM files for external processing to enhance transparency regarding data security and patient confidentiality.
10. The figures, particularly the ROC curves (Figure 1), are critical for understanding the software's performance. Consider adding interpretative comments directly on the figures or in the captions.
11. The comparison between sub-cohorts in Table 2 is a strong feature of this manuscript. However, is this a result of insufficient power for smaller groups? Would a larger study be able to identify more nuanced differences?
12. The discussion focuses heavily on the statistical results without fully addressing the clinical significance of the findings. For instance, while the newer software's AUC is statistically higher, what does this mean for TB screening programs in practical terms? Could these gains in AUC translate into earlier diagnosis or better case management?
13. The sensitivity and specificity trade-offs are important but need further elaboration. Discuss how recalibrating thresholds will impact operations.
14. Consider discussing the implications of this study for low- and middle-income countries in more detail. Could the increased diagnostic testing triggered by a higher sensitivity software version strain health resources, and how should programs prepare for this?
15. Standardize the abbreviation for "AUC" throughout the manuscript (sometimes referred to as "ROC AUC").
16. Ensure consistent use of hyphens and spacing, e.g., in "past-history" and "past history" of TB.
17. The median abnormality score data could be better visualized by adding a table for easy comparison across different subgroups.
