# Supplementary material for: Comparison of different Lunit INSIGHT CXR software versions when reading chest radiographs for tuberculosis
Source: PLOS Digit Health. 2025 Apr 15;4(4):e0000813. doi: 10.1371/journal.pdig.0000813 (PMC11999130; doi:10.1371/journal.pdig.0000813)
Supplement: Attachment — Submitted filename: Response to Reviewer Comments 2025-01-06.docx [file pdig.0000813.s002.docx]

**Journal Requirements:**

In the online submission form, you indicated that "The test library’s anonymized DICOM files may be shared upon a reasonable request to the corresponding author.". All PLOS journals now require all data underlying the findings described in their manuscript to be freely available to other researchers, either a in a public repository, within the manuscript itself, or uploaded as supplementary information.

The de-identified DICOM library has been uploaded to Dryad, and the manuscript has been updated with a permanent DOI link to the data repository. The data repository is currently only available to reviewers (via the below link), but will be made public if/when this manuscript is accepted for publication.

<http://datadryad.org/stash/share/nlaUohUFEeksF3A59h4CDjXTSEXrg4Yv6i7ojBF07jY>

In the online submission form, you indicated that your data will be submitted to the Dryad database upon acceptance. Should your submission be accepted, we will require the following information in your Data Availability Statement:

1. The DOI provided by Dryad

2. The citation for your data package in the reference section of your manuscript

3. The citation for your data package in the methods section

If you are unable to adhere to our open data policy, please kindly revise your statement to explain your reasoning and we will seek the editor's input on an exemption.

See the above response. In order to be accept to the Dryad data repository, we had to drop the HIV status variable from the data set in order to avoid the inclusion of more than three indirect identifiers (e.g. age group + sex + TB status + HIV status). We felt that this was acceptable since only five (0.2%) test library participants self-reported having HIV.

While revising your submission, please upload your figure files to the Preflight Analysis and Conversion Engine (PACE) digital diagnostic tool. If there are other versions of figure files still present in your submission file inventory at resubmission, please replace them with the PACE-processed versions.

Figures files were uploaded to PACE and files which meet the system’s standards were replaced in manuscript draft and were uploaded into the journal’s submission system.

**Reviewer #1**

Introduction: It might be beneficial to expand slightly on the limitations of previous CAD software studies to highlight the novelty of the current research.

The introduction states that no studies have been done with this particular CAD software.

Methods: It would be helpful to include more details on the ethical considerations and consent processes, particularly given the retrospective nature of the study.

Verbal consent was obtained during the programmatic ACF which served as the source data for this evaluation. This is standard practice as community CXR screening for TB is a service provided by the government within Ministry of Health screening guidelines. Only de-identified program data were used for analysis.

Results: Further elaboration on the potential clinical implications of these findings could be beneficial.

We have expanded on the clinical implications of the study in the discussion.

Discussion: Expanding on how these findings might influence TB screening protocols and CAD software deployment could provide additional value.

We have expanded on how the use of an un-calibrated software would impact TB screening programs.

Items to consider:

Ensure that all abbreviations are defined at first use.

The manuscript has been proofread and all abbreviations are defined when they are first used.

Correct any typographical or grammatical errors.

The manuscript has been proofread and all mistakes corrected.

Ensure that references are correctly formatted and relevant to the study.

Thank you for this feedback. All citations were reviewed and updates were made to ensure that the numbering, names and journal titles were correctly included in the manuscript. These edits were done using Zotero reference manager and do not show as track changes in the marked up manuscript.

**Reviewer #2:**

The summary abstract in the generated PDF (I assume where you enter the data in the online system is slightly different than the manuscript abstract and results – one mentions 10.4% and then in the manuscript 10.3% is the overall positivity rate for the sample.

We reviewed the manuscript text and abstract text in the submission portal to ensure that it consistently says 10.3% of test library participants have an Ultra positive result.

Lines 12-15 sometimes the new (better performing) AUC number is listed first and sometimes it is second, making it confusing for the reader. I would just list the newer and higher number first so it flows better.

The text has been standardized to always present the predecessor software version (3.1.0.0) and then the new software version (v3.9.1.0).

Line 50. We often talk about design locked products for diagnostics and I think what the authors are referring to is that the CAD products change more regularly than other TB diagnostics for example Xpert. However, Xpert MTB/RIF changed during the time it was produced – and each of those changes was a new design locked product. In the same vein, 3.1.0.0 and 3.9.0.1 are locked. They do not change based on their data they interpret, and they are CE marked for that specific version. It gives the wrong impression to a reader in the current wording and should be changed to something like, as opposed to laboratory-based diagnostics, CAD software is updated regularly with new versions going through separate regulatory approval... or something to that effect.

The text has been updated to better convey this message. Mentions of design locking have been removed.

Lines 52-53 – I generally agree with the statement but your reference 8 from Murphy et al actually showed that V4 was marginally better than V5 for AUC – although V6 outperformed them both.

The text has been updated slightly to more clearly indicate that the newest CAD software versions have significantly better performance than their predecessors, rather than each successive version have better performance.

Line 63 – positive should be with a lowercase p.

The text was updated.

65-66 you might consider mentioning how many older/younger individuals tested positive for TB in addition to the yield.

The text was updated to clearly indicate the number of people included in the DICOM test library and the number with MTB positive TB by gender.

82 I very much like the point about the performance gain in the lower levels of sensitivity which is quite important and not often discussed in CAD publications.

No changes required.

145-146. However, when an abnormality score cut-off threshold is set in order to maximize sensitivity, the new software version appears to be significantly less specific than its predecessor – i am not sure this manuscript is showing this to be true – as a comparison of the two software which leads me to my major suggestion for the submission. The analysis compares the performance of V3.9 at the same threshold that 3.1 achieved 90% sensitivity. This is a good comparison as it demonstrates the importance of understanding how versions differ. Some changes are small – V3 and 4 of qXR and some are very large as Fehr and colleagues pointed out with CAD4TB 5,6, and 7 and they have real consequences for implementers who do not pay attention to the changes. However, as a full evaluation the authors should make the comparison at the sensitivity level – not only same threshold. Although it is implied in the AUC mentioned above in line 82, and then again in 140 but it is not explicit. The authors do present improved in sub-populations in Table 2 which is also nice to show improvement but the same could be done with how AUC translates to people detected/tests conducted/saved. I think a simple table could show the reader if a program wanted to fix sensitivity at 90% for either version, what would it mean for performance gain/loss tests used etc. This is critical for implementers to keep in mind. Some providers are offering perpetual licenses. Often, to upgrade there may be cost implications. So does it make sense for a program to spend money to upgrade if the performance gain would be minimal? Especially if deploying on many sites. I understand the library is curated and not reflective – but the analysis and approach is the same and I suggest be presented and discussed to make the statement on lines 141-75 stronger and easier to interpret.

Thank you for this valuable feedback. Table 3 has been updated to include a column for change in Ultra testing and Row 4 has been dropped, in order to focus the message on the need for threshold calibration. The table now more clearly shows a significant change in specificity and follow-on testing indication if you upgrade the software and continue using the same threshold by comparing Rows 1 and 2. And then it shows no significant difference in specificity and Ultra testing indication when you re-calibrate the threshold by comparing Rows 1 and 3. Corresponding text in the results and discussion sections have been updated to better align with the more focused presentation of results.

148-150. I am not sure why humans are mentioned here since they are outside of the analysis and is pure supposition – both for the newer and older versions.

This text has been cut from the paragraph, so that the discussion flows into the paragraph about threshold setting more smoothly.

160-163. While I understand the point of the statement, I don’t think it is a strong one. Presumably, if as stated in lines 153-54 an implementer (ideally) would have gone through an analysis and evaluation of the software to arrive at a threshold score to select. The same implementer could use the same images to analyze with the new software. The point is that they must do it to ensure optimal performance, but I don’t think the statements in 153 and 160 can both be true. The authors should reinforce the importance of doing such analysis at before large scale implementation as well as with any software update and that developers should be more up front about the changes they make and how it could affect performance at different thresholds.

This text has been cut from the paragraph.

**Reviewer #3:**

The abstract needs to be rewritten. The objective of the study is not clearly stated and jumps directly into the contributions and results.

The abstract has been rewritten to better state the objective of the study and highlight the results.

The phrase "performance characteristics were compared at selected cut-off thresholds" is unclear—briefly explain why these thresholds were chosen.

The text has been updated to briefly define why those thresholds were selected.

The introduction needs more details on why comparing CAD versions is important for clinical practice, especially in resource-constrained settings. While the shortage of radiologists is mentioned, explain how the findings could directly impact health outcomes.

The third paragraph of the introduction has been updated to include additional text about the findings from other CAD software version analyses and why they are important for clinical practice.

Add more recent studies to highlight developments in CAD systems beyond the three mentioned systems (CAD4TB, INSIGHT CXR, qXR).

Text has been added to the end of the second paragraph to indicate that other CAD software for TB have been developed and independently evaluated; references to studies have been pulled up from the Discussion to this section. We do not feel it is necessary to discuss the CAD software landscape in further detail, as all published version analyses have been done with the WHO-recommended CAD software.

Add more recent studies on the clinical impact of CAD software in TB diagnostics within the field.

We have not updated the background with additional description of the clinical impact of CAD software. We feel that the citation of WHO guidelines, and the comparative CAD software evaluations cited, provide sufficient context for this technology. This manuscript’s focus is on the implications of version upgrades, not the primary use of the CAD software.

The limitations section should explain in more detail how using the Ultra test as a reference could bias the results. Although the issue of false positives in individuals with a past TB history is mentioned, expanding on the potential for this bias and its effect on the study’s conclusions is necessary.

The limitations paragraphs have been updated to better explain potential sources of bias and their impact on CAD software performance.

Highlight the need for prospective validation more clearly. The differences between study conditions and real-world settings should be expanded.

The second paragraph of the discussion has been updated to talk about the implications of an un-calibrated thresholds and the need for more prospective studies.

In the methods section, how does the current threshold calibration method (TDR or iterative score calibration) compare in terms of ease of implementation, costs, and time?

We believe this is outside the scope of the manuscript. We conducted a retrospective analysis which identifies a critical time point for when abnormality score cut-off thresholds should be reassessed. We did not design a test library to evaluate calibration methods.

Provide further details on the anonymization of DICOM files for external processing to enhance transparency regarding data security and patient confidentiality.

The text has been updated to better explain how DICOM files were de-identified.

The figures, particularly the ROC curves (Figure 1), are critical for understanding the software's performance. Consider adding interpretative comments directly on the figures or in the captions.

No edits to the ROC curves in Figure 1 have been made. We do not feel that this is standard practice among published CAD software evaluations. However, in the corresponding text in the results section, has been edited to more clearly highlight the region with performance gains.

The comparison between sub-cohorts in Table 2 is a strong feature of this manuscript. However, is this a result of insufficient power for smaller groups? Would a larger study be able to identify more nuanced differences?

We agree. Text has been added to the limitations paragraphs to describe challenges with interpreting the sub-cohort analyses.

The discussion focuses heavily on the statistical results without fully addressing the clinical significance of the findings. For instance, while the newer software's AUC is statistically higher, what does this mean for TB screening programs in practical terms? Could these gains in AUC translate into earlier diagnosis or better case management?

The relevant discussion paragraphs have been updated to discuss the possibility of improvements in detection for specific sub populations, and the clinical significant of the score changes.

The sensitivity and specificity trade-offs are important but need further elaboration. Discuss how recalibrating thresholds will impact operations.

The second paragraph of the discussion has been updated to cover this feedback.

Consider discussing the implications of this study for low- and middle-income countries in more detail. Could the increased diagnostic testing triggered by a higher sensitivity software version strain health resources, and how should programs prepare for this?

Text has been included to highlight that over testing may burden laboratories and reduce effectiveness of the program.

Standardize the abbreviation for "AUC" throughout the manuscript (sometimes referred to as "ROC AUC").

We have ensured that AUC is used in a standard way throughout the manuscript.

Ensure consistent use of hyphens and spacing, e.g., in “past-history” and “past history” of TB.

We have ensured consistent use of hyphens throughout the manuscript.

The median abnormality score data could be better visualized by adding a table for easy comparison across different subgroups.

The bar graphs have not been updated. We believe they clearly show shifts in the score distribution through their relative heights and labels.
