# Supplementary material for: Comparison of different Lunit INSIGHT CXR software versions when reading chest radiographs for tuberculosis
Source: PLOS Digit Health. 2025 Apr 15;4(4):e0000813. doi: 10.1371/journal.pdig.0000813 (PMC11999130; doi:10.1371/journal.pdig.0000813)
Supplement: Attachment — Submitted filename: Response to reviewer comments 2025-03-03.docx [file pdig.0000813.s003.docx]

**Response to the second round of reviewer comments**

**Additional Editor Comments (if provided):**

Thank you for revising this manuscript. Please see the remaining minor comments from Reviewer 3 about adding information on the clinical impact of CAD systems, and improving clarity. I believe addressing these would improve the manuscript.

The manuscript has been updated in line with the feedback from Reviewer #3 in Questions 4 and 6.

**Reviewers' Comments:**

1. If the authors have adequately addressed your comments raised in a previous round of review and you feel that this manuscript is now acceptable for publication, you may indicate that here to bypass the “Comments to the Author” section, enter your conflict of interest statement in the “Confidential to Editor” section, and submit your "Accept" recommendation.

Reviewer #2: All comments have been addressed

Reviewer #3: All comments have been addressed

No response required.

2. Does this manuscript meet PLOS Digital Health’s publication criteria? Is the manuscript technically sound, and do the data support the conclusions? The manuscript must describe methodologically and ethically rigorous research with conclusions that are appropriately drawn based on the data presented.

Reviewer #2: Yes

Reviewer #3: Yes

No response required.

3. Has the statistical analysis been performed appropriately and rigorously?

Reviewer #2: Yes

Reviewer #3: Yes

No response required.

4. Have the authors made all data underlying the findings in their manuscript fully available (please refer to the Data Availability Statement at the start of the manuscript PDF file)?

Reviewer #2: No

Reviewer #3: Yes

A quantitative data set and corresponding DICOM (chest X-ray image) files have been uploaded to the Dryad repository. A link was included in the manuscript draft which editors and reviewers can use to access data during the peer review process. I was advised by the Dryad repository editor to fully publish the data set once the manuscript has been accepted by a journal. In this manuscript revision, I have included the permanent Dryad DOI link (not yet active), in the hopes that it will soon be accepted and I can fully publish the data set.

Permanent Dryad DOI link (after publication):

<https://doi.org/10.5061/dryad.5tb2rbpcv>

Temporary Dryad link (for peer review): https://datadryad.org/share/nlaUohUFEeksF3A59h4CDjXTSEXrg4Yv6i7ojBF07jY

5. Is the manuscript presented in an intelligible fashion and written in standard English?

Reviewer #2: Yes

Reviewer #3: Yes

No response required.

6. Review Comments to the Author

Reviewer #2: (No Response)

Reviewer #3: The authors have made significant revisions that address most of the points raised. However, some areas, such as the inclusion of recent studies on the clinical impact of CAD systems, could be further expanded. Additionally, adding interpretative comments on figures and visualizing median scores in a table would improve clarity. Despite these minor suggestions, the manuscript has improved considerably, and I believe it is now suitable for publication with minor revisions.

More recent studies on the clinical impact of CAD software on TB screening were added into the introduction, including a 2025 comparison against multiple radiologists and key studies from India and Viet Nam showing increased yields and reduced diagnostic test consumption and radiologist workloads.

The original Figure 2 (bar graph) was dropped from the manuscript. Table 3 already shows the median Lunit CAD abnormality scores by software version and participant cohort. The methods and results text has been updated to describe the key finding of the original Figure 2, which is that a sizable proportion of people with TB still have a low Lunit CAD abnormality score, even when using the new software version.

7. Do the peer reviewers want their identity to be public for this peer review?

Reviewer #2: No

Reviewer #3: Yes: Farhana Yasmin
